# Improving Pallet Mover Safety in the Manufacturing Industry: A Bow-Tie Analysis of Accident Scenarios

**DOI:** 10.3390/ma11101955

**Published:** 2018-10-12

**Authors:** Karolien van Nunen, Paul Swuste, Genserik Reniers, Nicola Paltrinieri, Olga Aneziris, Koen Ponnet

**Affiliations:** 1 Research Chair Vandeputte, Law Enforcement, Faculty of Law, University of Antwerp, 2000 Antwerp, Belgium; 2 Antwerp Research Group on Safety and Security (ARGoSS), Engineering Management, Faculty of Applied Economics, University of Antwerp, 2000 Antwerp, Belgium; P.H.J.J.Swuste@tudelft.nl (P.S.); G.L.L.M.E.Reniers@tudelft.nl (G.R.); 3 Safety and Security Science, Faculty of Technology, Policy and Management, Delft University of Technology, 2628 CD Delft, The Netherlands; 4 Department of Mechanical and Industrial Engineering, Norwegian University of Science and Technology—NTNU, 7491 Trondheim, Norway; nicola.paltrinieri@ntnu.no; 5 National Centre for Scientific Research “Demokritos”, 15310 Agia Paraskevi, Greece; olga@ipta.demokritos.gr; 6 Media, ICT & Interpersonal Relations in Organisations & Society (MIOS), Faculty of Political and Social Sciences, University of Antwerp, 2000 Antwerp, Belgium; koen.ponnet@uantwerpen.be

**Keywords:** manufacturing industry, bow-tie analysis, pallet mover accidents, accident analysis, safety barriers

## Abstract

A Belgian manufacturing company uses pallet movers for internal transport. Despite the company’s efforts to improve occupational safety, accidents with pallet movers remain noteworthy. In order to control occupational accidents, it is crucial to have a clear view of the potential accident scenarios that are present in a company. The bow-tie method is a way to capture and visualize these accident processes in an integrative way. Included in the bow-tie are safety barriers (both technical as organizational and human) and management delivery systems that can intervene in these accident processes. Once bow-ties are composed, they are an excellent point of departure to assign indicators to the safety barriers and management delivery systems in order to control (i.e., prevent or mitigate) accident scenarios. Two types of indicators can be distinguished. Firstly, there are general indicators that are assigned to management delivery systems interrupting multiple accident scenarios, which can yield a higher safety gain (as they intervene in multiple accident scenarios). Secondly, there are scenario-specific indicators targeting one specific accident scenario, which can be valuable as they target a specific problem in the company. For the development of the bow-ties, a multi-method design with the inclusion of different data sources was used, leading to a comprehensive overview. This makes the bow-tie analysis of internal transport with pallet movers transferable to other settings where pallet movers are used for internal transport.

## 1. Introduction of the Study

Internal transport represents a well-known occupational hazard in many modern industrial environments. Pallet movers (synonyms for ‘pallet mover’ are pallet jack, walkie-rider, pallet truck, transpallet) are used for internal transport in several industries, and these machines are easy to operate, compared to, for example, forklift trucks. However, pallet movers are inherently dangerous machines. They often operate close to pedestrian workers and, charged with a load, their total mass can be well above two tons. Often, their load is not secured to the machine, leading to instability when gravity gets a grip of the load.

The company under investigation in this study is a manufacturing plant located in Belgium, which is part of an American multinational, producing consumer products all over the world. Pallet movers are used frequently for internal transport. Like many major American companies, this company also pays a lot of attention to the safety of their employees. The company uses the so-called 6W-2H and why-why techniques to analyze its recordable accidents without or with lost work time. The 6W-2H problem analysis produces a description of the context of accidents (what, where, which, when, who, to whom, how, how much). The why-why analysis is performed to get to the so-called root causes of accidents and is based on mapping of what happened during the accident process and why this happened. Countermeasures are formulated based on the results of these accident analyzes. The entire accident analysis is the responsibility of the supervisors, but are hereby supported by the Health and Safety (HSEQ) staff.

According to figures provided by the European safety manager of the abovementioned multinational, the 6W-2H and why-why techniques did not help in reducing occupational accidents substantially. As an alternative, a bow-tie analysis is proposed, providing a detailed and comprehensive insight into potential accident scenarios, including possible safety barriers (both technical and non-technical) and management delivery systems which can prevent or mitigate the accident processes. In Section 4, the choice for the bow-tie method is supported.

To test the bow-tie analysis, a pilot project was formulated, focussing on accidents during internal transport with pallet movers. Accidents with pallet movers represent a significant share of the total number of accidents. In the years 2015 and 2016, about ten percent of all recordable accidents with lost work time that occurred at the European plants of the multinational involved a pallet mover.

The research question of this study is ‘Which accident scenarios are possible during internal transport with pallet movers and which safety barriers (both technical as organizational and human) and management delivery systems can influence (i.e., prevent or mitigate) these accident scenarios?’.

## 2. Background of the Plant under Investigation

At the Belgian plant, approximately 300 people are employed. Two out of three employees are permanent (‘own company employees’) and one out of three is a contractor.

The production process is manifested on several floors. The upper floors are responsible for supplying raw materials, the production of intermediate products, and supplying these intermediate products to lower floors, where product finishing and packing take place. The ground floor has production lines for finishing the products and packing lines. These lines are implanted in an existing space which was initially not designed for that purpose.

The entire plant is characterized by a lot of load manipulation, which means that products are stocked in many temporary buffers. For example, on the ground floor, the packing materials are transported from the warehouse to a first large buffer (by a forklift truck), followed by transportation from the first large buffer to a second smaller buffer (by a pallet mover), and from the second smaller buffer to a buffer in front of the production or packing line (by a pallet mover).

The transportation routes of forklift trucks are separated from transportation routes of pallet movers, leading to (almost) no possible contact between the two types of internal transport.

**Figure 1:**
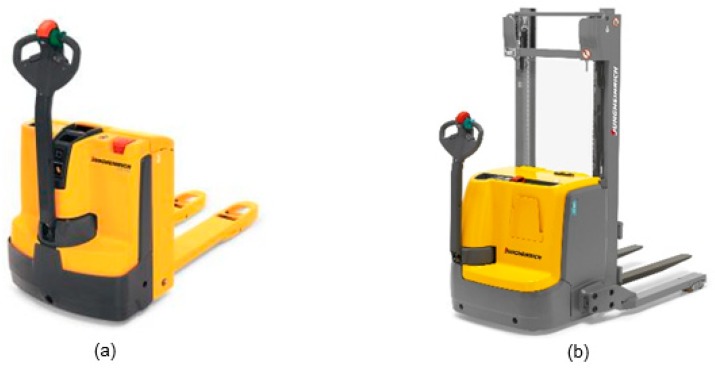
The types of pallet movers—(**a**): standard pallet mover and (**b**): stacker.

Figure 1 shows two types of pallet movers being used at the plant: Standard electrical pallet movers and electrical stackers which can lift loads to approximately 1.8 m. Pallet movers can transport a load of 1.2 to 2 tons, have a driving speed up to 6 km/h and a standard emergency stop. Maintenance and repair of pallet movers are performed by the company itself on a regular basis, while inspections are performed by an external company every three months.

Approximately 75% of the operators handling pallet movers are provided by one steady contractor company. The contractor staff is characterized by a frequent rotation.

**Figure 2:**
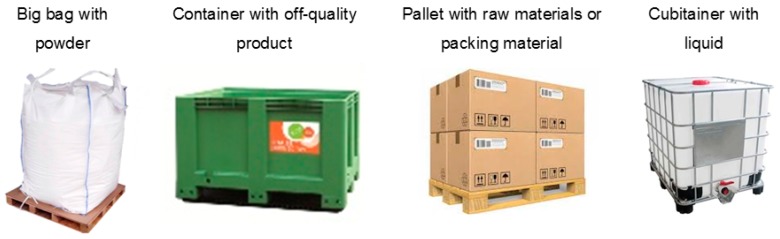
The most frequent loads transported with pallet movers.

Figure 2 shows the most frequent loads transported at the plant. Finished products are not transported with pallet movers, only raw materials, intermediate products, packing material, and off-quality products.

During the period from 2015 to 2016, eight recordable accidents occurred at the Belgian location. In three of these accidents, a pallet mover was involved. Two of these pallet mover accidents happened with a contractor employee, and one with a company employee.

## 3. Research Methodology

To compose the bow-ties of accidents with pallet movers, a multi-method design was used.

A literature search was conducted using electronic databases of the library of the Delft University of Technology, the British Health and Safety Executive (HSE), the American Occupational safety and Health Administration (OSHA), and the American National Institute for Occupational Safety and Health (NIOSH). Search terms were the following: ‘pallet mover’, ‘pallet jack’, ‘walkie-rider’, ‘pallet truck, ‘transpallet’, and ‘accident’. Safety related articles on pallet movers were rather scarce in the literature. Therefore, articles on forklift truck accidents (using the search term ‘forklift’) were also included in the literature study, for as far as the accident processes had similarities with the ones of pallet movers.

Belgian and Dutch national data on pallet mover accidents were requested. For Belgium, data on pallet mover accidents are obtained from Fedris, the Belgian federal agency for occupational risks. For The Netherlands, Storybuilder is used, which is a software tool developed for the Dutch Ministry of Social Affairs and Employment [1]. Both agencies have provided data on accidents reported to and investigated by the labor inspectorate. In both databases, the type of equipment involved in the accidents can be selected. This equipment classification is based on the classification by ESAW (European Statistics on Accidents at Work). Pallet movers fall under the code 11.04 (‘mobile handling devices, handling trucks (powered or not)—barrows, pallet trucks, etc.’). It should be noted that the data obtained does not only cover accidents with pallet movers, but also other equipment falling under the same code. Hence, national numbers of pallet mover accidents cannot be given. However, the databases also contain information on types of accident scenarios, root causes, and failing safety barriers. The latter qualitative information was used to complement the development of the bow-ties.

Documents and data concerning pallet mover safety available at the Belgian plant (and by extension at all European plants if available) were analyzed: The material of the pallet mover training (presentation, syllabus), an observation checklist used to evaluate the use of pallet movers (HSEQ staff and supervisors use this checklist to evaluate the behavior of operators during pallet mover use), the minutes of monthly safety meetings, a pallet mover maintenance checklist, the most recent pallet mover inspection overview, a checklist for interims regarding pallet mover use, and the safety notifications regarding pallet movers in the incident registration system. During 2015–2016, 127 safety notifications regarding pallet movers were available at the Belgian plant comprising information on accidents (*n* = 9), near-misses (*n* = 9), unsafe conditions (*n* = 93), and positive feedback to the operators (*n* = 16). All available accident analyzes (the 6W-2H and why-why techniques) performed after a recordable accident with a pallet mover were also taken into account. In Appendix A, an example of such a recordable accident is given, containing a short description of the accident and the actions taken in response to the accident.

At the Belgian plant, observations were being held at the workplaces of the pallet mover operators. A personal introduction of the researchers and the purpose of the study were given before the observations, and an introduction was given to the contractors during their daily team meetings. Observations were complemented with interviews with operators (*n* = 25), team leaders (*n* = 5), the HSEQ staff (*n* = 3), and management (*n* = 2). Interviews with operators and team leaders were performed on-the-job and took approximately fifteen minutes per person. The following aspects were addressed during the interviews:

- A job description of their tasks involving a pallet mover
- Problems and obstacles encountered during the use of pallet movers
- Facilitating aspects regarding the use of pallet movers
- Accidents, near-misses, or unsafe conditions with pallet movers
- Accidents that are most likely to occur with pallet movers
- Suggestions for improvement regarding the use of pallet movers
- Additional questions based on the observations

Interviews with the HSEQ staff and management were performed in a meeting room and took approximately one hour per person, discussing topics as the safety management system and findings during the fieldwork. The staff of the external company that provides the training of the pallet mover operators was also interviewed.

## 4. The Bow-Tie Model

**Figure 3:**
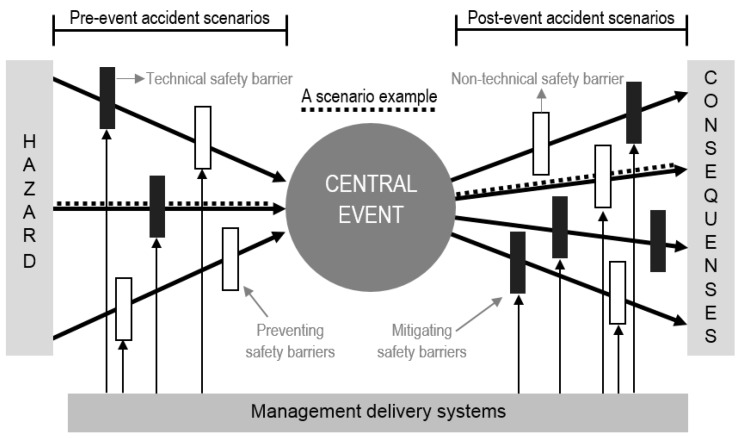
The bow-tie model.

The safety metaphor used in this study is the so-called bow-tie (Figure 3). The bow-tie model originates from the engineering domain and combines a hazard and safety barrier concept, dating as far back as DeBlois (1926), Gibson (1961) and Haddon (1963), together with a scenario concept, known from the Swiss cheese model of Reason (1997) [2,3,4,5,6,7,8]. A bow-tie model is comprised of a fault tree (the left-hand side of the model), which represents the risk factors of a failure, and an event tree (the right-hand side of the model), which represents the consequences of a failure [9].

The bow-tie metaphor illustrates an accident process, starting with a hazard on the left-hand side. A hazard (or energy) is a source or a condition with the potential for causing harm. Various accident scenarios, pictured as left-right arrows, can migrate to the center point of the metaphor, the central event. This central event represents a state where the hazard (energy) has become uncontrollable and, thus, becomes an undesirable event with a potential for harm or damage. The central event proceeds the consequences at the right-hand side of the metaphor, such as causing harm to people or damage to assets or environment.

The strength of the metaphor is its relationship between accident scenarios, technical safety barriers, non-technical safety barriers, and management delivery systems. A scenario is a sequence of events and conditions necessary for an accident to occur. Looking at the scenarios, two types of events can be distinguished. There are pre-event accident scenarios presented at the left-hand side of the central event (leading to the central event), and there are post-event accident scenarios depicted at the right-hand side of the central event (leading to the consequences). The technical safety barriers, represented as the black boxes in the scenarios, are technical entities that can interrupt the accident scenario. An example of a technical safety barrier is an emergency stop on a pallet mover. The non-technical (or organizational and human) safety barriers are represented as the white boxes in the scenarios, being non-technical entities that can interrupt the accident scenario. An example of a non-technical safety barrier is the removal of leaking cubitainers (which interrupts the pre-event accident scenario of losing control over the pallet mover due to leaked products on the floor). The upwards arrows in Figure 3 represent the influence of management delivery systems. Management delivery systems influence the quality (in terms of reliability and availability) of the technical and non-technical safety barriers. For example, maintenance of the emergency stop on the pallet mover does not interrupt the accident scenario in a direct way, but is a management delivery system influencing the reliability of the technical safety barrier ‘emergency stop on pallet mover’. Another example of a management delivery system is the training of pallet mover operators on removing leaking cubitainers, which influences the reliability of the non-technical safety barrier ‘removal of leaking cubitainers’.

There are two types of safety barriers that can be distinguished. There are safety barriers to prevent the occurrence of the central event, which are presented in the pre-event accident scenarios, and there are safety barriers to control or to mitigate the consequences, which are presented in the post-event accident scenarios.

The bow-tie model has a hidden time factor. Less than adequate safety barriers or management delivery systems can be manifested over a long period of time. If a hazard becomes uncontrollable and reaches the central event, scenarios reaching their consequences will usually unroll very quickly. Pre-event accident scenarios may take days, week, months, or even longer, while post-event accident scenarios develop in hours, minutes, or even shorter.

After bow-ties are developed, the next step is to assign indicators to the safety barriers and the management delivery systems [10]. Indicators are able to visualize possibilities for improvement, to indicate safety improvement or safety decline over time, and create benchmarking (e.g., between different plants). Once the indicators are developed, targets and limits should be assigned to every indicator (what is acceptable or unacceptable as a result or for instance tolerable with leeway for improvement). Additionally, responsibilities have to be set up in order to achieve goals and to define actions when targets are not met. According to the needs of the company, indicators can be prioritized (which indicators are for instance more important or more feasible to implement).

Traditionally, the scope of accident and incident investigations, whether performed internally or externally, is usually limited to investigating the immediate causes and decision making processes related to the accident sequence. Important factors contributing to the accident are hereby often overlooked. However, since the method used to analyze incident data and accident information influences the proposed prevention measures, it should be of no surprise that those investigations don not directly guide one towards the most effective improvements and solutions. On the other extreme, methods and models to investigate the socio-technical system, such as those proposed by Rasmussen (‘drift to danger’ model) [11], Leveson (the STAMP method) [12] or Hollnagel (the FRAM method) [13], are often too general for the application intention, and this way surpass their goal. The bow-tie method as employed in this research finds a way between both situations, and thoroughly investigates a (possible) accident without being too high-level.

The majority of accident modeling techniques has been designed to address process safety. However, most of these models tend to be also applicable to the field of occupational safety [14]. The same applies for the bow-tie model, which has entered the field of occupational safety through the European Workgroup for development of the Occupational Risk Model (WORM), which started with the aim of decreasing the occupational accident rate in the Netherlands by 10–15% [14,15]. The value of this model lies mainly in its suitability for qualitative analysis. In addition, bow-ties have been proposed for quantifying occupational risk in the framework of the WORM research project. An initial [16] and a more general form of the model have been presented in Reference [17]. Bowties for quantifying occupational risk have been presented in the following cases: Falls from heights [18], falling objects [19], contact with moving parts of machines [20], activities near moving vehicles [21], fires [22] and hazardous substances [23].

## 5. Results

### 5.1. The Outcomes of the Current Accident Analysis in the Plant under Investigation

At the Belgian plant, pallet mover accidents are being analyzed with the 6W-2H and why-why techniques. This accident analysis allows us to focus on technical, organizational and human aspects. However, in the accident analysis of the plant, a trend of ‘blaming the victim’ can be identified. For example, looking at the pallet mover accident as described in the Appendix A, the following aspects can be indicated as important contributors for the accident: The area was very crowded creating a narrow maneuvering space, the load that had to be picked up was not standing in the designated zone and the production pressure was high. The accident analysis shows that the involved operator was personally blamed, as disciplinary measures against the operator were taken by the contractor company.

The countermeasures that are taken based on the results of the accident analysis are also mainly directed towards the operators, such as giving personal warnings and the retraining of pallet mover operators. The observation checklist to evaluate the behavior of operators during the use of pallet movers is often used after an incident occurred, meaning that it is implicitly assumed that the behavior of the operator is one of the root causes of incidents. Organization-oriented countermeasures mainly focus on the adaptation of the working environment to create more maneuvering space and a better overview.

### 5.2. Bow-Ties of Accident Processes with Pallet Movers

Table 1 presents an overview of the left side of the bow-tie: Possible hazards, pre-event accident scenarios, preventing technical and non-technical safety barriers, preventing management delivery systems, and central events of accident processes with pallet movers. Table 2 comprises central events, mitigating technical and non-technical safety barriers, mitigating management delivery systems, post-event accident scenarios, and consequences.

Table 1 and Table 2 were composed based on the findings of the literature study [24,25,26,27,28,29,30,31,32,33,34,35,36,37,38,39,40], information available through Belgian and Dutch national accident databases, the analysis of documents and data available at the Belgian plant regarding pallet movers, and the results of the fieldwork (observations and interviews).

## 6. Assigning Indicators to the Bow-Ties

Once the bow-ties are composed, they are an excellent point of departure to assign indicators to the technical and non-technical safety barriers and to the management delivery systems in order to control (i.e., prevent or mitigate) the accident scenarios. Indicators can support a company’s safety management and provide information on a preferred safety goal.

It is important that the indicators focus on aspects that are applicable to the operating environment. For instance, it could be an improvement to replace all present pallet movers with new, less heavy equipment. However, in the context of the company, it is possible that this is not feasible due to budgetary constraints. Another example is the reorganization of the layout of the floors, which is an important aspect in order to create more maneuvering space. However, in the plant under investigation, installations are integrated into an existing space which was initially not designed for that purpose, leading to space constraints. Additionally, in the past years, several improvements have already been made regarding the layout of the working space and the work floor, and there is, of course, a limitation to the possibilities in creating more maneuvering space. A question arising from this space constraint is whether pallet movers are the best equipment to use at particular spaces in a production facility with limited maneuvering space and if it would not be better to search for an alternative way to transport the material. This is related to the inherent safety of a company [41], which will be discussed at the end of this section.

Besides the applicability of indicators in the company environment, the focus of the indicators can also be chosen based on the presence of management delivery systems. For instance, as can be seen in Table 1 and Table 2, certain management delivery systems are present in multiple scenarios. It concerns the following management delivery systems: ‘training of pallet mover operators’, ‘sensitization and communication’, ‘guidelines and procedures’, and ‘planning of production and staffing’. Indicators assigned to these management delivery systems can be considered as general indicators, as they are linked to multiple accident scenarios. As an example, the management delivery systems ‘training of pallet mover operators’ is further elaborated below.

Next to the general indicators, scenario-specific indicators can also be developed. Decisions on what scenarios should be focused on can be based on the plant-specific risks regarding pallet mover use. Two examples of company-specific pre-scenarios will be further elaborated below, being ‘narrow maneuvering space’ and ‘leaking cubitainers’.

### 6.1. General Indicators: Training of Pallet Mover Operators

In Table 3, the possible indicators for the frequently occurring management delivery system ‘training of pallet mover operators’ have been elaborated. An indicator of the content of the (re)training is proposed, and an indicator of the quality control of the (re)training. Additionally, there are indicators regarding the coverage ratio of the training and the retraining.

It should be noted that a certain sequentiality is present in the follow-up of the indicators. For instance, a high coverage ratio of the (re)training is negligible if the content of the (re)training is not tailored to the needs of the company and if the quality of the (re)training is evaluated as substandard.

### 6.2. Scenario-Specific Indicators: Narrow Maneuvering Space

**Figure 4:**
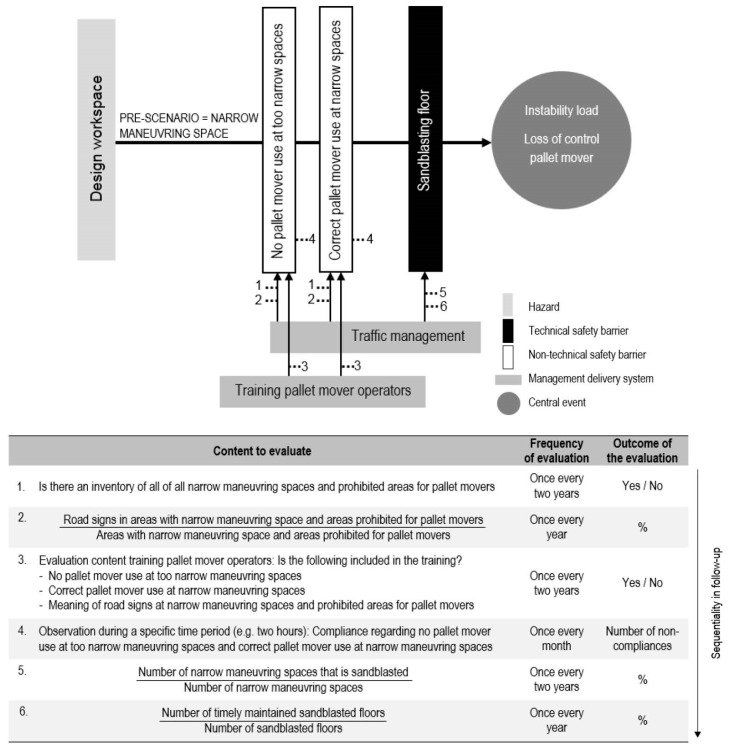
The possible indicators for the pre-scenario of ‘narrow maneuvring spaces’.

**Figure 5:**
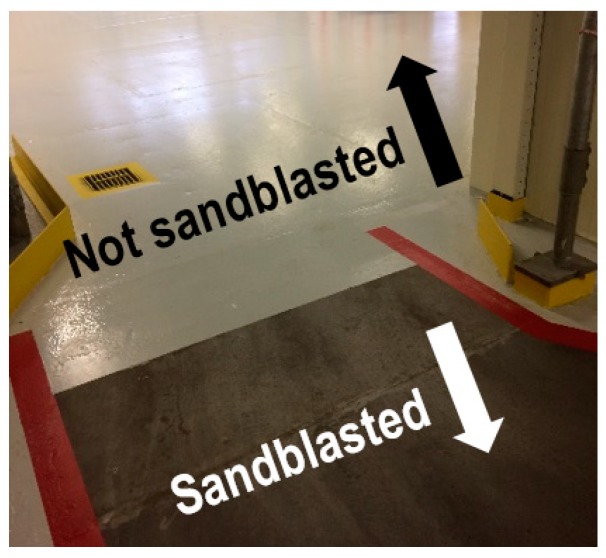
An example of a sandblasted and not sandblasted pallet mover transportation route.

A specific risk at the plant under investigation is the narrow maneuvering space. Therefore, this scenario was chosen to be further elaborated into scenario-specific indicators. Figure 4 shows the bow-tie of the pre-event accident scenario of a narrow maneuvering space. Only a selection of possible safety barriers and management delivery systems has been included in the bow-tie. Two non-technical safety barriers have been included: ‘no pallet mover use at too narrow spaces’ and ‘correct pallet mover use at narrow spaces’. One technical safety barrier has been included: ‘sandblasting floor’. Sandblasting of the transportation routes with pallet movers (Figure 5) leads to a better grip and a shorter breaking distance. The following management delivery systems have been included: ‘traffic management’ and ‘training pallet mover operators’.

As with the general indicators, a sequentiality is also present in the follow-up of the scenario-specific indicators. For example, when a company does not include correct pallet mover use at narrow maneuvering spaces in the training, it does not make sense to evaluate non-compliances on this topic.

### 6.3. Scenario-Specific Indicators: Leaking Cubitainer

**Figure 6:**
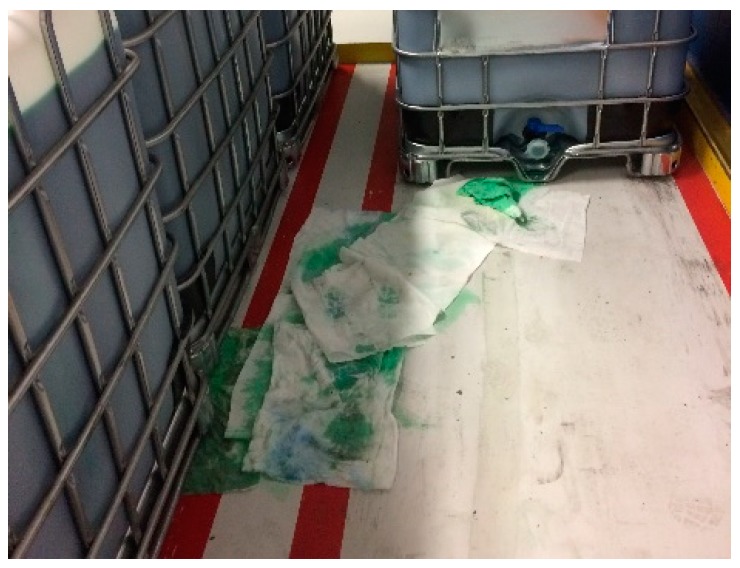
A cubitainer with leaking drain valves leading to a spill of the liquid content.

**Figure 7:**
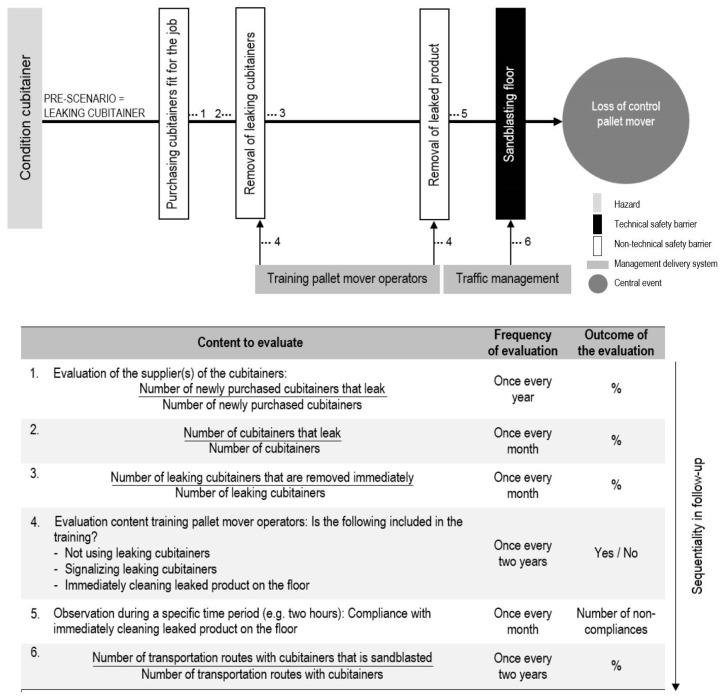
The possible indicators for the pre-scenario of ‘leaking cubitainer’.

Another specific risk at the plant under investigation is the worn drain valves of cubitainers, leading to spills of liquid content (Figure 6). Products on floors lead to a longer breaking distance and a higher chance of losing control over the pallet mover. Figure 7 presents the bow-tie of the pre-event accident scenario of damaged loads, and more specific leaking cubitainers, complemented with possible indicators for this specific scenario. Again, only a selection of possible safety barriers and management delivery systems has been included in the bow-tie, namely, the non-technical safety barriers ‘purchasing cubitainers fit for the job’, ‘removal of leaking cubitainers’, and ‘removal of leaked product’, the technical safety barrier ‘sandblasting floor’, and the management delivery systems ‘training pallet mover operators’ and ‘traffic management’.

### 6.4. Evaluation of the Indicators

Once the indicators have been determined, they have to be evaluated. Based on the specificity of the indicator and the needs of the company, this evaluation can take place on a yearly basis, or if needed with a lower or a higher frequency.

Additionally, after the development of the indicators, targets and limits have to be assigned to every indicator. This means that the company has to decide what is acceptable as a result and what is not. In the example of the coverage ratio of the training, a target could be that 100% of all pallet mover operators should be trained for pallet mover use. In the example of the number of leaking cubitainers, a target could be that <5% of the cubitainers is leaking.

An important aspect is that responsibilities have to be indicated: Who does what and when in order to reach the goals of the indicators. In the example of the (re)training, responsibility has to be indicated for the subscriptions for the (re)training. The same applies to responsibilities to take actions when a target is not achieved.

Based on the elaborated examples, it could seem that a lot of collection and registration is needed for all indicators. However, it should be noted that the given examples and their accompanying indicators are very specific. Once the entire set of indicators has been developed, it will become clear that many of them can be collected and registered under the same heading.

In order to facilitate an adequate monitoring of the indicators, a system should be set up to report and to collect the required data. Such systems are often already (partly) present in a company.

To be complete, something should be said on the necessity of the indicators, which is a reflection that should be made before developing the indicators. After all, it should first be analyzed if the processes that are present in the company are as inherently safe as possible [41]. In the given example regarding the narrow maneuvering spaces, whether a pallet mover is the safest equipment to use at these places (and by extension at all places) should be considered. In the example of the leaking cubitainers, whether cubitainers are necessary and if the process cannot be designed in such a way that the transportation of the liquid is minimalized (and by extension that a large amount of manipulation of all loads at the plant is minimalized) should be considered. Kletz’s [42] article entitled “what you don’t have, can’t leak” is a good resume of this matter.

## 7. Discussion and Conclusions

The outcomes deriving from the current accident analysis that is used at the Belgian plant under investigation (6W-2H and why-why technique) does not seem sufficient to take adequate measures in order to prevent accidents with pallet movers. When preventive measures are taken based on accident analyses, several shortcomings can be identified. Firstly, preventive measures based on accident analyzes only focus on accidents that already occurred, leaving all other potential accident scenarios out of scope. A method that includes not only company specific data, but also generic data sources such as the literature and national accident data, generates information on the entire accident process, including aspects that have not (yet) occurred at a specific plant. Additionally, it can be concluded that the recommendations resulting from the current accident analysis that is used at the plant under investigation, are mostly individual-oriented. This is however not an intrinsic problem of the 6W-2H and why-why technique, as this technique focusses on both technical, organizational as human aspects. Hence, the focus on the human aspects is not a consequence of the technique itself, but of the way the technique is applied.

To address the shortcomings of the current accident analysis, an alternative method—i.e., the bow-tie method—is chosen in order to address pallet mover safety. The bow-tie was chosen for several reasons. Firstly, the bow-ties were composed using a multi-method design. This multi-method design leads to a better comprehensiveness of the entire accident process of pallet mover use and gives a detailed overview of what could possibly go wrong with a pallet mover. In the bow-ties, the possible causes and consequences of potential accidents are identified. Additionally, the bow-tie includes the influence of safety measures (safety barriers and management delivery systems) on the evolution of accident scenarios [43].

Due to the use of a multi-method design to compose the bow-ties, not only company specific data were included, but also generic data sources such as the literature and national accident data. This leads to information on the entire accident process, including aspects that have not (yet) occurred at a specific plant.

Because of the comprehensive character of the bow-tie method, the results are easily transferable to other production facilities where pallet movers are used for internal transport, assuming that the hazards are the same. This means that, if this study was conducted in another production facility with similar hazards and similar a working environment, composition of the bow-ties would have led to a similar outcome as in Table 1 and Table 2 (this does not mean that the process of linking indicators to the bow-ties is the same, as this is very company specific). In other words, the bow-tie method leads to a general model that is transferable and applicable in every setting where, in this case, pallet movers are being used. However, the indicators may be different.

Another advantage of the bow-tie method is that it allows us to make a clear distinction between preventing and mitigating safety barriers and management delivery systems.

Seven hazards regarding pallet mover use could be identified based on the composition of the bow-ties: Load, the speed of the pallet mover, acceleration of the pallet mover, the design of the workplace, conditions of the workplace, conditions of materials (load and pallet mover), and operating the pallet mover. Through several identified pre-event accident scenarios, these hazards can lead to different central events: Instability of the load, loss of control over the pallet mover, and a breakdown of the pallet mover. At their turn, these central events can lead through several post-accident scenarios to different consequences: Injury, damage, or economic loss. Several technical and non-technical safety barriers and management delivery systems to prevent or mitigate the central event could be linked to the accident scenarios.

The identified safety barriers and management delivery systems mainly focus on organizational aspects, and, to a lesser extent, on the individual behavioral aspects of operators. The pitfall of ‘blaming the victim’, which is often present in other methods of accident analysis where there is primarily focused on the individual behavior of the operators, is therefore reduced when using the bow-tie method.

Once bow-ties are composed and safety barriers and management delivery systems have been identified, indicators should be developed and monitored consequently. These indicators should be composed based on their applicability in the company, meaning what is possible given a specific company environment. When developing indicators, an important distinction can be made. Firstly, there are general indicators. In the bow-ties, certain management delivery systems can be linked to many of the accident scenarios. When indicators are developed for frequently occurring management delivery systems, these indicators can be considered as general because they are not linked to only one scenario. Secondly, there are scenario-specific indicators. This means that indicators are linked to specific scenarios that require attention in a plant. With both the general and the scenario-specific indicators, a certain sequentiality should be acknowledged in the follow-up of the indicators. For all indicators, it is therefore important to set priorities.

To conclude, indicators are an important result of a bow-tie analysis. When a company reaches a consensus on a set of indicators to be monitored, a unique insight is obtained on the status and development of potential accident scenarios. Management can intervene adequately to ensure a safe production.

## Figures and Tables

**Table 1:** The elements on the left-hand side of the bow-ties of accident processes with pallet movers based on: ^(1)^ Literature; ^(2)^ Belgian and Dutch National data; ^(3)^ Document analysis; ^(4)^ Safety notifications in the incident registration system of the Belgian plant; ^(5)^ Accident analysis; ^(6)^ Fieldwork: Observations and interviews.

Hazard	Pre-Event Accident Scenario	Preventing Technical and Non-Technical Safety Barriers and Management Delivery Systems	Central Event
Load ^(1,2,3,4,5,6)^	Limited sight due to height of load ^(1,2,3,5,6)^ Load not secured/not well secured (e.g., no cage, not strapped) (e.g., due to production pressure) ^(1,2,3,4,5,6)^ Load wrongly loaded (e.g., not centered, too highly loaded, forks under width pallet instead of length, containers not straight on each other, too heavy) ^(2,3,4,5,6)^	Camera on pallet mover ^(1)^ Mirror in workplace ^(1,2,6)^ Traffic management: floor markings ^(1,2,3,5,6)^ Use horn on pallet mover ^(1,2,3,4,5,6)^ clothing employees with reflective clothing ^(2)^ Design pallet mover (less heavy) ^(1)^ Bump detection ^(1,3,6)^ Loading correctly (e.g., no load on double pallets) ^(2,3,4,6)^ Secure the load (e.g., stretching, strapping, cage) ^(1,2,4,5,6)^ Provision of material to secure the load ^(6)^ Using the material to secure the load ^(2,4,6)^ Clear and accessible guidelines/procedures for loading ^(3,6)^ Training pallet mover operator ^(1,3,4,5,6)^ Sensitization/communication on loading ^(3,4,5,6)^ Planning production and staffing (sufficient time and people to perform the job) ^(1,5,6)^	Unstable load ^(1,2,3,4,5,6)^ Loss of pallet mover control ^(1,2,3,4,5,6)^
Speed pallet mover ^(1,2,3,4,6)^ Acceleration pallet mover ^(1,6)^	Driving or accelerating too fast ^(1,2,3,4,6)^ Due to production pressure Wanting to take a break faster Due to gross negligence (play behavior, act tough)	Speed limitation ^(1)^ Acceleration limitation ^(1,6)^ Emergency stop pallet mover ^(1,6)^ Provision of a sufficient number of pallet movers ^(3)^ Clear and accessible guidelines/procedures on speed and acceleration ^(3,5,6)^ Training pallet mover operator ^(1,3,4,5,6)^ Sensitization/communication on speed and acceleration ^(3,4,5,6)^ Planning production and staffing (sufficient time and people to perform the job) ^(1,5,6)^	Unstable load ^(1,2,3,4,5,6)^ Loss of pallet mover control ^(1,2,3,4,5,6)^
Design workspace ^(1,2,3,4,5,6)^	Limited sight due to insufficient light ^(1,2,6)^ Distraction due to hard/insufficient noise ^(1,5)^ Too narrow of a maneuvring/parking space ^(1,2,3,4,5,6)^ Limited sight at crossings, corners, entrances/exits ^(1,3,4,5,6)^ Ridges, sharp endings at infrastructure (e.g., at pallet lift, diked areas) ^(4,5,6)^ Too crowded working area/buffers (e.g., too much load, too much traffic) ^(2,4,5,6)^ Pedestrian or other internal transport in the zone of the pallet mover transport ^(2,4,5,6)^ Unsafe location to recharge thebattery ^(4,6)^ Unsuitable floor material (too rough or too smooth) ^(2,6)^	sup>∙ Sufficient light ^(1,6)^ sup>∙ Ear protection ^(6)^ Camera on pallet mover ^(1)^ Mirror in workplace ^(1,2,6)^ Sandblasting floor ^(6)^ Traffic management: Floor markings ^(1,2,3,5,6)^, stop sign or other road signs ^(1,2,4,6)^, separate pathways and lanes ^(1,2,6)^ Use horn on pallet mover ^(1,2,3,4,5,6)^ Bump detection ^(1,3,6)^ (Reorganisation) layout (e.g., sufficient pre-defined zones for full and empty pallets or cubitainers, as least load manipulation as possible) ^(1,2,3,4,5,6)^ Increasing the frequency of emptying buffers ^(3,4,5,6)^ Shielding ridges, sharp endings at infrastructure ^(6)^ Clear and accessible guidelines/procedures (e.g., for maneuvring in a narrow space) ^(3,5,6)^ Training pallet mover operator ^(1,3,4,5,6)^ sup>∙ Provision of predefined locations to recharge the battery ^(6)^	Unstable load ^(1,2,3,4,5,6)^ Loss of pallet mover control ^(1,2,3,4,5,6)^
Condition workspace ^(1,2,3,4,5,6)^	Floor in bad condition (e.g., holes, loose floor plates) ^(1,2,3,4,6)^ Insufficient housekeeping (e.g., loads outside predefined spaces, pallets not stacked correctly) ^(2,3,4,5,6)^ Insufficient cleanliness (e.g., wet floor, the products on the floor) ^(2,3,4,5,6)^ Incoming rain, humidity of the workspace due to condensation ^(6)^	Supervising housekeeping and cleanliness ^(3,4,5,6)^ Cleaning wet/dirty floor ^(4,5,6)^ Shield of damages and repair as soon as possible ^(6)^ Sandblasting floor ^(6)^ Lift the forks 10–20 cm while driving to avoid irregularities in the surface ^(3)^ Clear and accessible guidelines/procedures (e.g., on housekeeping) ^(3,5,6)^ Training pallet mover operator ^(1,3,4,5,6)^ Sensitization/communication (e.g., on importance of notifications of damaged floor, on importance housekeeping) ^(3,4,5,6)^	Unstable load ^(1,2,3,4,5,6)^ Loss of pallet mover control ^(1,2,3,4,5,6)^
Condition material (equipment and load) ^(1,2,3,4,5,6)^	Poor condition pallet mover due to absent maintenance (e.g., malfunctioning emergency stop) ^(1,2,3,4,6)^ Not inspected pallet movers ^(3,4,6)^ Damaged load (e.g., damaged pallets, leaking cubitainers) ^(1,3,4,6)^ Misfit between pallet mover and load ^(1,4)^ Substantial mass of pallet mover ^(1)^ Pallet mover or load not fit for the job (e.g., pallet mover too wide for the workspace) ^(3,4,5)^	Sandblasting floor ^(6)^ Purchasing pallet movers that are fit for the job ^(3,4,5)^ Purchasing less heavy pallet movers ^(1)^ Purchasing loads that are fit for the job ^(3,4)^ Timely and adequate maintenance of pallet mover ^(3,4,6)^ Timely and adequate inspection of pallet movers and loads ^(3,4,5,6)^ Timely replacement of outdated pallet movers ^(3,5,6)^ Removing damaged loads ^(1,3,6)^ Provide inspection mark that is not obstructive during work (e.g., sticker instead of a strap) ^(4,6)^ Communication with the supplier of damaged loads ^(3)^ Performing start-up check of pallet mover (e.g., of an emergency stop) ^(3,4,5,6)^ Providing clear/readable start-up checklist that is easy to fill in ^(3,4,6)^ Clear and accessible guidelines/procedures (e.g., on start-up check pallet mover) ^(3,5,6)^ Training pallet mover operator ^(1,3,4,5,6)^ Sensitization/communication (e.g., on not using damaged pallets, on signalising defects at pallet movers) ^(3,4,5,6)^	Unstable load ^(1,2,3,4,5,6)^ Loss of pallet mover control ^(1,2,3,4,5,6)^ Breakdown pallet mover ^(1,2,3,4,5,6)^
Operating the pallet mover ^(1,2,3,4,5,6)^	Not following the safe practices for pallet mover use ^(2,3,4,5,6)^ Due to ignorance or inexperience ^(3,5,6)^ Due to production pressure ^(3,4,5,6)^ Due to distraction operator (e.g., using a phone, reading during pallet mover use) ^(2,4,5)^ Due to gross negligence (intoxication, play behavior, act tough) ^(2,3)^ Not following the traffic rules (e.g., ignoring a stop sign) ^(4,5)^ Losing control due to a physical/medical problem (e.g., fatigue, bad eyes, concentration impaired by medication ^(2,3)^ Operator in a no-go area for pallet movers ^(1,4,5,6)^ Unauthorised use of pallet mover (no or an expired licence) ^(3,4,5,6)^ Difficult maneuvring situations (e.g., pallet needs to be turned first) ^(6)^ Non-routine circumstances (e.g., maintenance machines) whereby the usual routes cannot be taken ^(4,6)^	Pre-selection of pallet mover operators ^(3,5,6)^ Training pallet mover operator ^(1,3,4,5,6)^ On-the-job assistance/training of new employees ^(5,6)^ Clear and accessible guidelines/procedures for pallet mover use (also for non-routine circumstances) ^(3,5,6)^ Sensitization/communication (e.g., on no-go areas) ^(3,4,5,6)^ Leadership supervision, coaching, and feedback on unsafe practices ^(3,4,5,6)^ Planning production and staffing (sufficient time and people to perform the job) ^(1,5,6)^ Sufficient predefined parking spaces for all pallet movers ^(3,4,5,6,6)^ Provide personal keys ^(3,4,6)^ Remove key from pallet mover when not in use ^(2,3,4,6)^ Take measures so it cannot be bypassed to start/drive pallet mover without keys ^(3,4,6)^ Always carry driving license for pallet mover ^(3,4,6)^ Visual and physical demarcation of no-go areas for pallet movers ^(6)^	Unstable load ^(1,2,3,4,5,6)^ Loss of pallet mover control ^(1,2,3,4,5,6)^

**Table 2:** The elements on the right-hand side of the bow-ties of the accident processes with pallet movers based on the ^(1)^ Literature; ^(2)^ Belgian and Dutch National data; ^(3)^ Document analysis; ^(4)^ Safety notifications in the incident registration system of the Belgian plant; ^(5)^ Accident analysis; ^(6)^ Fieldwork: observations and interviews.

Central Event	Mitigating Technical and Non-Technical Safety Barriers and Management Delivery Systems	Post-event Accident Scenario	Consequence
Unstable load (1,2,3,4,5,6)	sup>∙ Application of first aid ^(2,4,5)^ On time ^(2)^ With the correct diagnose and response action ^(2)^ sup>∙ Personal Protective Equipment (PPEs) ^(2,5)^ Provision of PPEs ^(2)^ Using the PPEs ^(2)^ Maintenance of PPEs ^(2)^ Safety features on pallet mover (e.g., tilt protection, bump detection) ^(2,3,6)^ Protection of objects/infrastructure (e.g., with foam, with steel bar) ^(3,5,6)^	Loss of load ^(1,2,3,4,5,6)^	Injury ^(1,3,4,5,6)^ Damage ^(1,3,4,5,6)^ Stop production process ^(4,5,6)^
Loss of control pallet mover ^(1,2,3,4,5,6)^ Breakdown pallet mover ^(1,2,3,4,5,6)^	Traffic management: Separate pathways and lanes ^(1,2,6)^ Emergency stop pallet mover ^(1,6)^ Increased visibility and/or audibility of pallet mover ^(2)^ Application of first aid ^(2,4,5)^ On time ^(2)^ With the correct diagnose and response action ^(2)^ sup>∙ Personal Protective Equipment (PPEs) ^(2,5)^ Provision of PPEs ^(2)^ Using the PPEs ^(2)^ Maintenance of PPEs ^(2)^ Safety features on the pallet mover (e.g., tilt protection, bump detection) ^(2,3,6)^ Protection of objects/infrastructure (e.g., with foam, with steel bar) ^(3,5,6)^	Pallet mover hits pedestrian/other internal transport ^(1,2,3,4,5,6)^ Pallet mover hits operator pallet mover ^(1,2,3,4,5,6)^ Pallet mover hits object/infrastructure ^(1,2,3,4,5,6)^	Injury ^(1,3,4,5,6)^ Damage ^(1,3,4,5,6)^ Economic loss (because of a stop of the production process) ^(4,5,6)^

**Table 3:** Possible indicators for the management delivery system ‘training of pallet mover operators’.

Sequentiality in follow-up	Content (re)training	Evaluation of the content of the (re)training every two years: Is the (re)training completely tailored to the needs of the company? (yes/no)Aspects to take into consideration: - Use of examples of specific risks and possible accident scenarios at the company? E.g., narrow maneuvring spaces, wet floors, too crowded buffers… - Sufficient rules and guidelines for the target audience?
	Quality control (re)training	Yearly evaluation of the percentage of participants of the (re)training evaluating the training as positively (i.e., a score of 7 out of 10 or higher)
	Coverage ratio training	Monthly evaluation of the percentage of starting pallet mover operators that are trained for pallet mover use
	Coverage ratio retraining	Yearly evaluation of percentage pallet mover operators receiving a retraining every five years

## Appendix A

**Table A1:** An example of a recordable accident with a pallet mover at the Belgian plant (input based on the 6W-2H and why-why analysis).

Description Accident Based on the Accident Analysis	Actions in Response to the Accident
An operator (contractor) got his foot clamped under the pallet mover, causing him to trip and pull the pallet mover over his foot, resulting in a contusion of his foot A load was urgently needed in order to avoid a line stop, so the operator broke with normal procedures and did not first remove the loads stocked in front of the load that had to be picked up Therefore, he had to pass through a narrow space and did not follow the safe practice of walking beside and not in front of the pallet mover The operator also did not follow the safe practice of keeping the pallet mover at arm’s length There were too many loads stocked by the forklifts in the area, creating the narrow space (the forklift truck driver responsible for the stocking of the area was not consulted during the accident analysis). The load that had to be picked up was not standing in the designated zone	Disciplinary measures against the operator were taken by the contractor company Communication on the accident and safe practices Retrain pallet mover operators Reorganization layout of the zone: a part of the loads is now stocked elsewhere, in order to create more maneuvring space
